# Spatiotemporal visual statistics of aquatic environments in the natural habitats of zebrafish

**DOI:** 10.1038/s41598-023-36099-z

**Published:** 2023-07-25

**Authors:** Lanya T. Cai, Venkatesh S. Krishna, Tim C. Hladnik, Nicholas C. Guilbeault, Chinnian Vijayakumar, Muthukumarasamy Arunachalam, Scott A. Juntti, Aristides B. Arrenberg, Tod R. Thiele, Emily A. Cooper

**Affiliations:** 1 Herbert Wertheim School of Optometry & Vision Science, University of California, Berkeley, CA USA; 2 Department of Biological Sciences, University of Toronto, Scarborough, ON Canada; 3 Werner Reichardt Centre for Integrative Neuroscience, Institute of Neurobiology, University of Tübingen, Tübingen, Germany; 4 Graduate Training Centre for Neuroscience, University of Tübingen, Tübingen, Germany; 5 Department of Cell and Systems Biology, University of Toronto, Toronto, Canada; 6 Department of Zoology, Department of Zoology, St. Andrew’s College, Gorakhpur, Uttar Pradesh India; 7 Department of Zoology, School of Biological Sciences, Central University of Kerala, Kasaragod, Kerala India; 8 Department of Biology, University of Maryland, College Park, MD USA; 9 Helen Wills Neuroscience Institute, University of California, Berkeley, CA USA; 10 Present Address: Centre for Inland Fishes and Conservation, St. Andrew’s College, Gorakhpur, Uttar Pradesh India

**Keywords:** Neuroscience, Sensory processing, Visual system

## Abstract

Animal sensory systems are tightly adapted to the demands of their environment. In the visual domain, research has shown that many species have circuits and systems that exploit statistical regularities in natural visual signals. The zebrafish is a popular model animal in visual neuroscience, but relatively little quantitative data is available about the visual properties of the aquatic habitats where zebrafish reside, as compared to terrestrial environments. Improving our understanding of the visual demands of the aquatic habitats of zebrafish can enhance the insights about sensory neuroscience yielded by this model system. We analyzed a video dataset of zebrafish habitats captured by a stationary camera and compared this dataset to videos of terrestrial scenes in the same geographic area. Our analysis of the spatiotemporal structure in these videos suggests that zebrafish habitats are characterized by low visual contrast and strong motion when compared to terrestrial environments. Similar to terrestrial environments, zebrafish habitats tended to be dominated by dark contrasts, particularly in the lower visual field. We discuss how these properties of the visual environment can inform the study of zebrafish visual behavior and neural processing and, by extension, can inform our understanding of the vertebrate brain.

## Introduction

The images cast on animals’ retinas during natural behavior have strong statistical regularities, which mold the structure and function of the visual system^1,2^. As such, substantial progress has been made in visual neuroscience by studying the properties of ensembles of images and videos of the natural world (see^3^ and^4^ for reviews). However, little is known about the unique visual demands for aquatic species used in neuroscience research.

Zebrafish in particular are a popular model animal in visual neuroscience, due to their diversity of visually-mediated behaviors already at larval stages, their relatively small brain size for vertebrate species, and their amenability at the larval stage for non-invasive imaging of neural activity throughout the central nervous system^5^. Advances in understanding visual processing in the zebrafish brain are broadly useful because they can inform our understanding of visual processing in vertebrate brains in general. Zebrafish are native to South Asia and are typically found in shallow water bodies with little to no flow^6^ or in slow flowing, small streams and rivers^7^. Aquatic environments like these differ from terrestrial habitats in notable ways. For example, particulates in the water interact with reflected light and likely reduce overall visual contrast. Snell’s window and the reflective water surface present different constraints on the content in the upper visual field as compared to terrestrial spaces. In addition, wave-induced light fluctuations occur underwater, and depending on wave dynamics and water depth, can create flicker and motion signals that have long been hypothesized to drive visual adaptations^8–11^.

Aquatic environments likely have strong statistical regularities that can shed light on our understanding of the cells and circuits that underlie visually-guided behaviors in these animals. Indeed, a range of recent work has suggested that properties of zebrafish vision and behavior can be explained by adaptations to specific visual patterns in their natural habitats^12–16^. Critical to further understanding zebrafish neural and behavioral adaptations to their environment is a determination of the spatiotemporal properties of contrast and motion in their habitat. Here we report the statistical analysis of a calibrated video dataset from the natural habitat of wild zebrafish. We compare these videos to a dataset of proximate terrestrial environments to identify unique visual demands of the aquatic environment that likely shaped how zebrafish encode and respond to visual cues.

## Materials and methods

### Field recordings

Data were analyzed from a video survey of zebrafish habitats in the Indian state of Assam over a two-week period in October 2019. The details of the video recordings, the devices used, and their calibration can be found in the methods section of^16^, which reports on the collection of the full dataset and describes a separate analysis of optic flow when the recording devices were moved to simulate swimming. Here, we focus on a detailed analysis of videos captured from stationary devices optimized for understanding the spatiotemporal visual statistics of the habitats. In brief, omni-directional recording devices (Insta360 One X) were positioned at a set of pre-selected sites in the natural range of zebrafish. The devices, which consisted of two fish-eye cameras, were housed in a waterproof case during video recording both underwater and in air and positioned using either a small tripod or a custom boom rig. Videos were recorded at 100 frames per second using the h.264 codec (MP4 file format) and frames were extracted as PNG image files (1504 × 1504 pixels for each camera).

Uncompressed video recording would have been preferable for an analysis of spatiotemporal statistics, because video compression algorithms tend to create blocking artifacts that produce illusory edges. However, the requirements for the field work and the components of the full recording apparatus necessitated the use of compressed recordings. By carefully processing the videos and focusing on relative comparisons between aquatic and terrestrial environments, we believe our analysis is largely robust to compression-related artifacts. For example, blocking artifacts are unlikely to strongly affect the large-scale contrast statistics that are the focus of many of our analyses, because these artifacts tend to affect local clusters of pixels and we average over large regions of the video frames. To reduce spatial artifacts, we also pre-filtered frames with a Gaussian smoothing kernel (σ = 2 pixels). Upon visual inspection, we observed a minor remaining temporal compression signature which resulted in small and transient reductions in mean luminance after every 100 video frames. As these reductions were very small (< 1% of mean luminance) we did not attempt to filter them out further. Our analysis of the spatiotemporal power spectra of the videos in the Fourier domain may be most susceptible to compression artifacts. However, the fact that we find different results in the spatial and temporal domains suggests that these results are due to the underlying scene statistics rather than the recording compression. Thus, we suggest that the compromises associated with video compression are reasonable in the context of the current analyses, but should be taken into account when interpreting the results.

Videos for this analysis were acquired from 9 sites across central Assam. Data from 3 sites were excluded due to positional instability of the camera and insufficient lighting. Table S1 and Fig. S1 provide locations and descriptions for the 6 sites included in our analysis (see Supplemental Material). At some sites, terrestrial videos were captured by positioning the camera within a natural scene near an aquatic sampling location or directly above the water. In total, 36 candidate videos were recorded from the 6 sites. Of these videos, 15 were excluded because the camera was transiently moving during recording, and one was excluded due to low light. This left 20 videos for further analysis (14 aquatic and 6 terrestrial).

### Video sampling

We subsampled the video dataset in both space and time. To sample spatially, four visual regions were taken from each video. Regions were centered on either -38° or 38° in azimuth relative to the center of each of the two fisheye lenses at an elevation of 0°. Each sample was a square region subtending 75 × 75°. Videos were typically a few minutes long. For the aquatic videos, we sampled the final 10 s of each recording to minimize the impact of the camera placement underwater. For the terrestrial environments, we performed the same spatial sampling, but re-sampled the same videos twice at two non-overlapping time points: the first (after experimenters were out of camera view) and the final 10 s of each video. This adjustment was made due to the comparatively smaller data set for terrestrial habitats. Following the methods described in^16^, estimates of the intrinsic properties of each camera were made using the Computer Vision Toolbox in Matlab and were used to rectify each region via perspective projection onto a plane orthogonal to the visual axis. For this analysis, we used only the green channel of the three-channel videos. Pixel intensity values were linearized using calibrated light measurements, such that they represent changes in light intensity that are linear with respect to the light levels in the environment. All details of the camera spatial intrinsics, spectral responses, and nonlinearities are included in a set of calibration files in the publicly available dataset, and are also reported in detail in the methods of the manuscript that originally introduced this dataset^16^.

**Figure 1:**
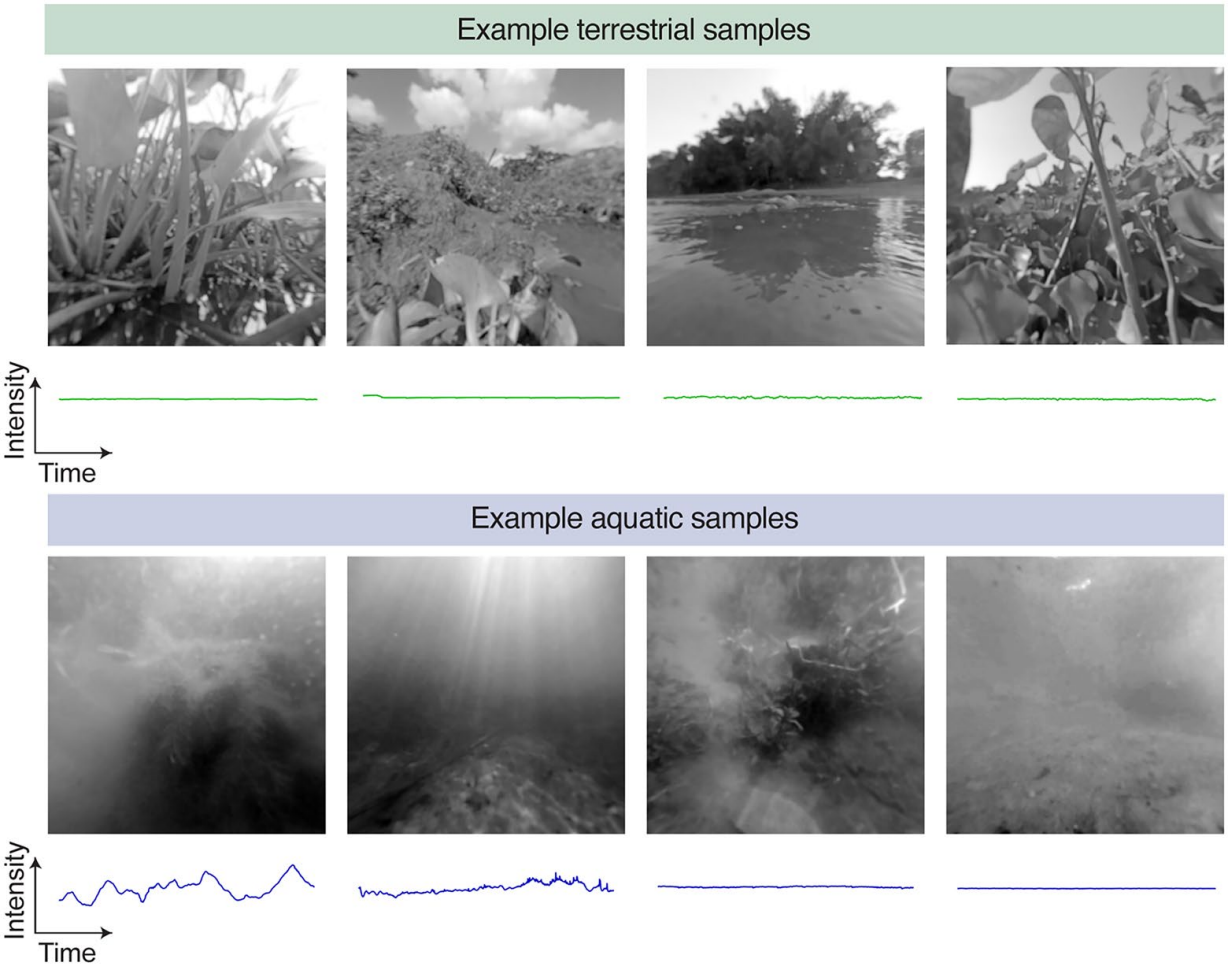
Examples of the first frame and the average change in intensity over time from the terrestrial and aquatic video samples. Each image shows the green (G) channel of one sample's first frame. The images are shown as log light intensity for visibility. Below each image is the average intensity as a function of time for the same sample. For all temporal plots, the abscissa is time with a span of 10 s, and the ordinate is intensity on an arbitrary scale.

Some samples were excluded from further analysis upon manual inspection. Pre-analysis exclusion criteria include: humans or equipment visible (n = 15), bubbles or debris attached to the dive case (n = 11), and excessive pixel saturation (n = 17). With respect to pixel saturation, limitations in camera dynamic range can lead to clipping in either the dark or the bright regions of a scene. To ensure that our measures of scene statistics were not biased by these artifacts, for each sample we calculated the percentage of pixels with intensity values falling within the bottom and top 2% of the maximum intensity value of the camera. Any sample with 10% or more pixels in these clipping ranges was excluded from analysis. Of the remaining samples, the clipped pixels were excluded from the contrast analysis (≈ 1% of pixels). Note that the spatial extent of all samples omitted Snell’s window. These exclusions left 25 terrestrial and 36 underwater samples for analysis. The first frames of several samples are shown in Fig. 1. Below each frame, the average intensity is plotted as a function of time. Videos showing all 61 samples are included in the Supplemental Material (Videos S1 and S2).

### Video analysis

#### Global contrast

We first wanted to examine the distribution of global visual contrast in the zebrafish habitat. We defined the global contrast (*C*) at each pixel location (*x*,*y*) to be

1 $$C(x,y) = \frac{{I(x,y) - \overline{I}}}{{\overline{I}}}$$

where *I*(·) denotes the raw light intensity, *x* and *y* denote the coordinates of each pixel, and *Ī* denotes the average intensity across all pixels within a given sample (75 × 75° over 10 s). This normalization accounts for differences in overall light levels across the different sites, which are not of primary interest in the current investigation because phototransduction processes adapt visual responses relative to the mean light levels (e.g.^17^). With this calculation, the mean contrast of each distribution is zero by definition (although the removal of saturated pixels can slightly alter the mean). We computed the variance, skewness, and kurtosis of global contrast across all pixels for each sample. We also calculated the probability density over linearly spaced bins ranging from contrast levels of −1 to 4.

#### Entropy

We used these global contrast measurements to calculate the entropy (*H*) of each sample, which provides a measure of the information carried by a signal. We calculated entropy from discrete probability distributions with *n* = 256 different levels (corresponding to 8 bits in the image), as follows:

2 $$H = - \sum\limits_{i = 1}^{n} {p(C_{i} ) \cdot \log_{b} [p(C_{i} )]} ,$$

where *p*(·) denotes the discrete probability density function, and *C*_*i*_ is the *i*-th level of global contrast. Here we measure entropy in bits (log base is *b* = 2). Intuitively, the products of the probabilities of each intensity level with the associated log probability collectively indicate the expected value of self-information: the amount of information in each level scaled by the probability of that level occurring.

#### Local contrast

We calculated local contrast at each pixel via locally-normalized difference of Gaussian filters. We convolved each video frame with a two-dimensional, zero-sum isotropic difference of Gaussians (*g*(*x*,*y*;*σ*), *σ*_center_ = 1°, *σ*_surround_ = 2°). We normalized each filter response by the local mean luminance, which was defined as the response of a unit-sum two-dimensional isotropic Gaussian with standard deviation equal to the surrounding portion of the contrast filter. Specifically, the local contrast (*C*_*L*_(*x*, *y*)) was defined as

3 $$C_{L} (x,y) = \frac{{I(x,y) * \left[ {g(x,y;\sigma_{center} ) - g(x,y;\sigma_{surround} )} \right]}}{{I(x,y) * g(x,y;\sigma_{surround} )}},$$

where ∗ is the convolution operator. Edge artifacts were removed before computing summary statistics.

#### Spatiotemporal power spectrum

To examine how visual contrast was distributed over space and time, we computed the spatiotemporal power spectrum of each sample. We can consider each video sample as a 3D data array consisting of two spatial dimensions (225-by-225 pixels) and one temporal dimension (1001 frames). We applied a 3D Hanning window to each sample to minimize edge artifacts, subtracted the mean value, and then performed a 3D Fast Fourier Transform. We grouped the Fourier signal amplitude into logarithmically-spaced spatiotemporal bins. In spatial frequency, the bins ranged from 0.05 cpd up to 0.75 cpd in 19 steps. In temporal frequency, the bins range from 0.5 to 50 Hz in 19 steps. These ranges were determined based on the spatial resolution of the videos, the camera frame rate, and the dimensionality of the video samples. Spatial bins were structured to capture all orientations equally (“rings” in Fourier space). We computed the mean power (square of the Fourier amplitude) as a function of spatial and temporal frequency separately by marginalizing the full power spectra across either dimension. These marginalized spectra were fit to a linear model. We also computed the joint spatiotemporal spectrum by calculating the mean power separately in each spatiotemporal frequency bin (361 bins total).

### Estimation of the spatial and temporal limits of zebrafish vision

**Figure 2:**
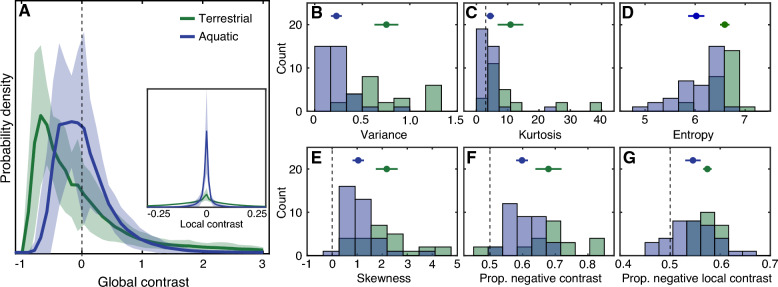
Contrast distributions differed between the terrestrial and aquatic samples. (**A**) Solid lines show the mean probability density of global contrast in terrestrial (green) and aquatic (blue) samples. The shaded area indicates the region plus or minus one standard deviation of the mean. (**B–G**) Statistics of the contrast distributions for each environment are plotted as histograms, including the variance (**B**), kurtosis (**C**), entropy (**D**), skewness (**E**), proportion negative global contrast (**F**) and proportion negative local contrast based on the difference of Gaussians contrast operator (**G**). Symbols above indicate the mean and standard deviation. Dashed lines indicate kurtosis = 3 (Gaussian), skewness = 0 (symmetric) and proportion negative contrast = 0.5. The inset in panel (**A**) shows the distribution of local contrast, which is much broader for the terrestrial samples. Note that the mean global contrast is always effectively zero because contrast is defined relative to the mean intensity of each sample, and is thus not included in the plots.

We wanted to examine this spatiotemporal spectrum in the context of the established limits of larval zebrafish vision. We thus compared this spectrum to spatial and temporal limits associated with optomotor responses (OMR), optokinetic responses (OKR), and prey capture responses. This analysis is visualized in Fig. 5A. For the temporal sensitivities of the OMR and OKR, we adopt approximate limits of 14 cycles/second^18,19^ and 2 cycles/second^20^, respectively. Note that the temporal limit for these behaviors depends on the tested spatial frequencies and velocities. The spatial limit (visual acuity) for the OKR has been reported to be 0.16 cycles/degree^21^, but estimating the spatial limit for the OMR was more complex. An OMR tuning curve to spatial frequency has been previously reported (Fig. 2a of^22^), showing that the OMR was drastically reduced for spatial frequencies of 0.08 cycles/degree or higher presented on a display. However, an aquatic visual stimulus setup with flat water container surfaces was used in this study, which introduces stimulus distortions such that the stimulus appears different to the fish than intended by the experimenter^23,24^. Based on the estimated distance from fish to water container bottom (0.25 cm, personal communication Jiaheng Xie), the distance from water container to stimulus screen (5.65 cm) and the estimated thickness of the water container and transparent stage (0.5 cm) in this study, we calculated the spatial frequency visible to the fish at the nadir based on the equations in Appendix 1 of Dunn et al.^23^. A stimulus grating 12° in spatial period (extending from the nadir) appears to the fish as a spatial period of 9.3°. We therefore adopt 10.8 cycles/degree as the visual acuity for OMR behavior. For prey capture behaviors, the spatial and temporal stimulus bounds are approximately 0.09 to 0.33 cycles/degree and 2 Hz to 60 Hz, respectively^25^. We also include a comparison with the the flicker fusion rate (20 Hz)^26,27^ and the theoretical cone spacing limit (0.24 cycles/degree) for larvae^21^.

### Statistical analyses

Pertinent features of the video samples selected for analysis were approximately normally distributed, so we adopted parametric statistics to examine differences between terrestrial and aquatic samples with a significance threshold of p < 0.05. Throughout the results, we use unpaired two-tailed t-tests and adopt d-prime as a measure of effect sizes. For the analysis comparing the upper and lower visual fields, we conducted 2 × 2 ANOVAs (terrestrial versus aquatic habitat; upper versus lower field) and conducted follow-up tests of pair-wise comparisons using the Tukey–Kramer method. Effect sizes for the ANOVAs are reported as partial eta-squared (η_p_^2^). For all statistics in the main results section, we report sample means, effect sizes, and p values. The full results, including test statistic values, degrees of freedom, and standard deviations, are reported in the Supplemental Material Tables S2 and S3.

## Results

### Zebrafish are relatively unlikely to encounter high contrasts in their habitats compared to terrestrial environments

A primary source of behaviorally-relevant visual information in the environment is contrast: variations in the relative brightness or darkness of points across space and time. For example, a predator approaching from above may block light and produce a looming region of relative darkness in the upper visual field; a potential food source may reflect a specularity that is brighter than the surrounding area^12,28–30^. We thus begin with an analysis of the overall distribution of contrast (Fig. 2).

Previous studies of terrestrial environments have shown several common features of how contrast is distributed: it tends to be unimodal with a peak near zero and leptokurtotic (i.e., heavy-tailed)^31–38^. These features are well-predicted by a basic model of the physical interaction between light and surfaces in the natural world in air^39^. Our terrestrial samples were consistent with this prior work. Figure 2A shows the average contrast distribution for the terrestrial samples and the standard deviation (green line and green shading). These distributions tended to be unimodal and heavy-tailed. In our sample, the modal contrast was less than zero (i.e., dominated by dark contrasts), which is not unusual (see next Section)^31,33^.

Similar to these terrestrial environments, the aquatic zebrafish habitat samples had an average contrast distribution that was unimodal and relatively leptokurtotic (Fig. 2A, blue line and blue shading). However, the shapes of the distributions from the two types of environments were clearly different. We found that the variance was lower in the aquatic habitats (Fig. 2B, M_Terrestrial_ = M_T_ = 0.75, M_Aquatic_ = M_A_ = 0.23, d′ = 2.03, p ≪ 0.001), as was the kurtosis (Fig. 2C, M_T_ = 11.00, M_A_ = 4.50, d′ = 0.83, p = 0.001). Variance and kurtosis are both measures of symmetric spread about the mean, so together these results suggest that high contrast points are overall less likely in zebrafish habitats. However, in both habitats the kurtosis was significantly greater than a normal distribution (p_T_ ≪ 0.001, p_A_ = 0.023), denoting the presence of heavy tails (low probability, high contrast features).

For a given measurement (say, contrast level, neuronal spike rate), entropy provides a means of quantifying how informative that measurement is on the basis of its probability distribution. For example, a variable whose distribution is an impulse function has minimum entropy—measurements are not needed to know what the value will be in a given instance, because it is always the same. A uniform distribution has maximum entropy because all levels are equally likely. We found that the samples from the zebrafish habitat had significantly lower entropy than the terrestrial samples, consistent with the observation that the probability mass is more concentrated towards zero contrast and the variance and tails are weaker (Fig. 2D, M_T_ = 6.60, M_A_ = 6.02, d′ = 1.46, p ≪ 0.001).

Taken together, these results suggest that high contrast features are relatively unlikely during natural visual experience in the zebrafish habitat. Behaviorally relevant information, however, can be conveyed by low probability, high contrast events. Importantly, the region of the visual field analyzed here does not include Snell’s window, which is much brighter than the rest of the visual field and potentially contains different contrast. Nonetheless, within the region of the visual field analyzed here, these results suggest that fewer neural resources in the zebrafish brain may be specialized for discriminating between levels of high contrast than would be predicted based on conventional terrestrial visual statistics.

### Zebrafish habitats are dominated by dark contrasts, similar to terrestrial environments

Another notable feature of natural contrast distributions from terrestrial environments is their positive skew^31,37–39^. That is, these distributions tend to have a long tail towards bright contrasts. Previous research examining visual sensation in terrestrial species has recognized that this positive skew leads to a preponderance of dark contrasts—that is, negative contrast coming from points with intensity below the local mean—during natural vision. Across a range of terrestrial species and levels of the visual hierarchy, the visual system appears to be adapted to exploit this dark dominance. These adaptations range from having smaller and more numerous OFF cells in the retina^37,40,41^ to cortical and subcortical receptive fields biased for encoding levels of dark contrast^42^ to motion processing mechanisms that exploit asymmetric luminance histograms^43,44^. Psychophysical studies in humans suggest that these neural biases have perceptual consequences, resulting in higher sensitivity to detect and discriminate patterns with dark contrast^45–48^.

Since the zebrafish visual system shares many of the basic structures for early visual encoding present in terrestrial species, it is important to examine whether a dominance of environmental “darks” may similarly play a role in shaping their visual systems. In our dataset, the aquatic contrast statistics indeed tended to be positively skewed (Fig. 2E, M_A_ = 1.00, p ≪ 0.001). This positive skew resulted in substantially more than half of the pixels having an intensity below the mean intensity (M_A_ = 0.60, p ≪ 0.001, Fig. 2F). Recall that we defined global contrast in this analysis relative to a large spatial region (75° square). When we calculated local contrast based on a smaller contrast operator, this dark bias was still present in the aquatic samples (Fig. 2A inset, Fig. 2G, M_A_ = 0.55, p ≪ 0.001). These observations are in line with recent findings suggesting that the zebrafish visual system contains asymmetric processing of bright and dark contrasts^13,14^, which may reflect adaptations to a dominance of visual information contained in dark contrasts similar to terrestrial species. Consistent with prior observations, the nearby terrestrial samples were also dark biased (Fig. 2E–G, skewness: M_T_ = 2.20, p ≪ 0.001; proportion negative global contrast: M_T_ = 0.68, p ≪ 0.001; proportion negative local contrast: M_T_ = 0.57, p ≪ 0.001). However, we note that the asymmetry was weaker in the aquatic habitats based on all measures (skewness: d′ = 1.23, p ≪ 0.001; proportion negative global contrast: d′ = 0.99, p ≪ 0.001; proportion negative local contrast: d′ = 0.79, p = 0.006). A weaker dark dominance in aquatic habitats may have quantitative implications for how ON and OFF pathways are structured to encode visual contrast in aquatic and terrestrial animals^49^. However, systematic differences in ON/OFF pathway organization have not yet been examined between animals living in different ecological niches.

### The dominance of dark contrasts in the zebrafish habitat is primarily in the lower visual field

**Figure 3:**
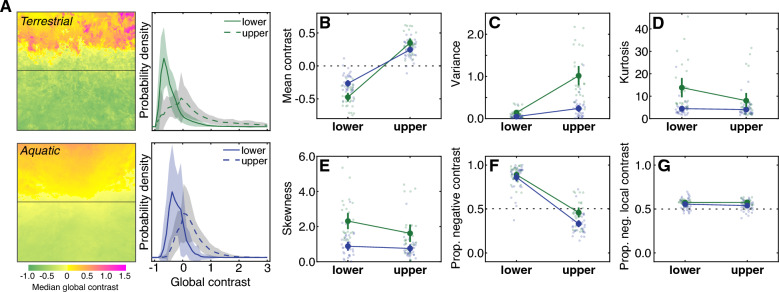
Contrast distributions differ in the upper and lower visual fields of both environments. (**A**) Heatmaps show the median contrast across the visual field for the terrestrial (upper) and aquatic (lower) samples. Line plots show the average probability density of contrast in the upper and lower visual fields. Shaded regions indicate plus or minus one standard deviation of the mean. (**B**–**E**) Statistics of the contrast distributions, including the mean (**B**), variance (**C**), kurtosis (**D**), skewness (**E**), proportion negative global contrast (**F**) and proportion negative contrast based on the difference of Gaussians local contrast operator (**G**). Values for individual samples are plotted as smaller circles; mean and 95% confidence intervals are plotted as large circles and vertical lines.

Several properties of visible light vary systematically as a function of elevation in the visual field, in both terrestrial and aquatic imagery^12,16,31,50,51^. These variations suggest that natural environments can place different demands on cells and circuits that typically encode the upper and lower visual fields. Upper/lower visual field asymmetries are prevalent in the zebrafish retina and in their behavioral responses^12,13,15,16,52^. Thus, we next examined the shapes of the contrast distributions above and below the midline (elevation = 0). Figure 3A shows the median contrast across the visual field for the terrestrial and aquatic samples, as well as the full distributions of contrast separated out for the upper and lower hemifields. We focus this analysis on main effects of visual field location (upper vs. lower) and interactions with environment (terrestrial vs. aquatic), since the main effects of environment were consistent with the results discussed in the previous section.

In both the terrestrial and aquatic samples, the mean contrast in the upper visual field tended to be higher than in the lower visual field (Fig. 3B). Because global contrast was normalized by the mean intensity across the whole visual field, this difference indicates that the upper visual field tended to be brighter and the lower visual field tended to be darker in both environments, which makes sense because in both environments illumination tends to come from above. There was a significant main effect of hemifield (upper vs. lower: M_up_ = 0.29, M_low_ =  − 0.35, η_p_^2^ = 0.83, p ≪ 0.001) and a significant interaction effect with environment (η_p_^2^ = 0.21, p ≪ 0.001). The interaction indicated that the bright contrast bias in the upper visual field did not differ significantly between aquatic and terrestrial samples (p = 0.05), while the dark contrast bias in the lower visual field was stronger in the terrestrial samples compared to aquatic (p ≪ 0.001).

As discussed in the previous section, the variance and kurtosis of contrast provide measures of symmetric spread. We found that variance tended to be higher in the upper visual field of both habitats (Fig. 3C). Again, there was a significant effect of hemifield (upper vs. lower: M_up_ = 0.56, M_low_ = 0.09, η_p_^2^ = 0.44, p ≪ 0.001) and a significant interaction (η_p_^2^ = 0.24, p ≪ 0.001). In the upper field, the variance was significantly higher in the terrestrial than the aquatic samples (p ≪ 0.001), but there was no significant difference in the lower visual field (p = 0.63). Examining higher order symmetric spread (kurtosis, Fig. 3D), we observed a main effect and interaction in the opposite direction (upper vs. lower: M_up_ = 5.70, M_low_ = 8.30, η_p_^2^ = 0.05, p = 0.015; interaction: η_p_^2^ = 0.04, p = 0.034). In the upper visual field, the kurtosis was not significantly different between the sample types (p = 0.12), but it was significantly higher in the terrestrial habitats in the lower visual field (p ≪ 0.001). Note that this finding extends our previously published analysis, which showed that contrast sparsity also varies as a function of elevation in these habitats^16^. Overall, the upper and lower visual fields were more similar to each other in the aquatic samples than in the terrestrial samples, suggesting that spatial variations are present in these environments but less robust.

We also found that skewness was more positive in the lower field in both environments (Fig. 3E; upper vs. lower: M_up_ = 1.10, M_low_ = 1.50, η_p_^2^ = 0.04, p = 0.02). While this visual field difference was descriptively stronger in the terrestrial samples, we did not observe a statistically significant interaction. In terms of the overall proportion of negative global contrasts in the upper and lower visual fields (Fig. 3F), there was a significant effect of hemifield (upper vs. lower: M_up_ = 0.38, M_low_ = 0.87, η_p_^2^ = 0.78, p ≪ 0.001) and a significant interaction (η_p_^2^ = 0.04, p = 0.037). The proportion of negative contrasts was higher in the lower visual field in both the terrestrial and aquatic samples (0.89 and 0.86, respectively; no difference between environments, p = 0.81). All other pairwise comparisons were statistically significant (p ≤ 0.001). When contrast was calculated based on the local operator, there was no longer a main effect of visual field or an interaction (upper vs. lower: M_up_ = 0.55, M_low_ = 0.56, η_p_^2^ = 0.01, p = 0.43; interaction: η_p_^2^ = 0.01, p = 0.37; Fig. 3G). This finding suggests that the extra lower field dark dominance primarily results from the low frequency bright to dark gradient created by the illumination from above (Fig. 3A), rather than from local differences in reflectance and shadows. Taken together, these results indicate that the upper and lower visual fields place consistently different demands on the zebrafish visual system in terms of encoding achromatic visual contrast. However, these differences are not as pronounced as in the terrestrial comparison samples. It is important to note that the separation into upper and lower visual field in this analysis assumes that the horizontal meridians of the animal’s eyes are aligned to the horizon, which is not always the case.

### Spectral power in zebrafish habitats is concentrated at lower spatial frequencies and higher temporal frequencies

**Figure 4:**
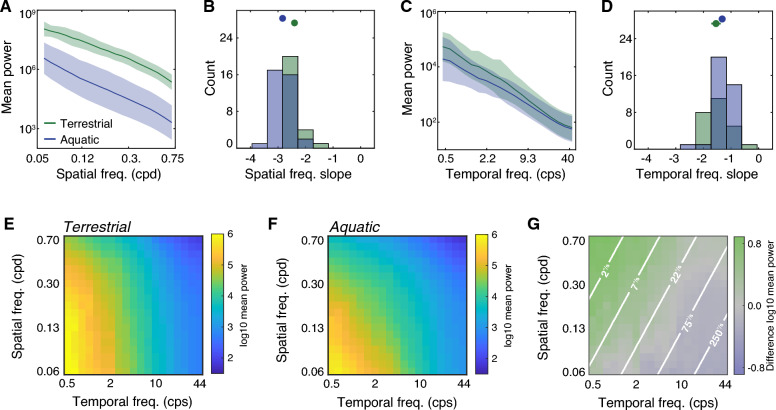
Spatiotemporal power spectra indicate more fast speeds in the aquatic samples. (**A**) Spatial power spectra. Lines indicate the mean log power across samples and shaded regions indicate plus or minus one standard deviation. Green and blue colors indicate terrestrial and aquatic samples, respectively. Cpd denotes cycles/degree. (**B**) Histograms of the best fit slopes of spatial power spectra in (**A**). The markers and errorbars show the means and the 95% confidence intervals. (**C**) Temporal power spectra, plotted in the same manner as (A). Cps denotes cycles/second. (**D**) Histograms of the best fit slope of temporal power spectra in (**C**). (**E**) The median terrestrial spatiotemporal power spectrum. (**F**) The median aquatic spatiotemporal power spectrum. (**G**) The difference between logarithmic values in (**E**) and (**F**). Lines illustrate iso-speed contours derived from the ratio of temporal frequency and spatial frequency.

The images cast on the retinas in natural environments exhibit complex spatiotemporal dependencies not captured by aggregated global or local contrast measures. These dependencies can be exploited by the visual system to further achieve efficient coding objectives. For example, images and video of terrestrial scenes are correlated in space and time: points that are nearby both spatially and temporally tend to be more similar in intensity and color^35,53–55^. These correlations manifest in a specific way in terrestrial imagery, which is revealed when natural images and video are analyzed in the frequency domain. Specifically, imagery of terrestrial environments peaks in power at low spatial and temporal frequencies and falls off roughly with a 1/f^n^ shape, where f denotes frequency and n is approximately 2. These data are often plotted in the log–log domain, leading to a linear function with a slope of -n. A range of theories have been proposed for how this pattern arises from physical properties of the environment, such as object occlusions and depth scaling^55–57^. Our samples from terrestrial imagery were consistent with this prior work. The spatial and temporal power spectra of the terrestrial samples are plotted as green lines in Fig. 4A and C, respectively. We fit the spatial and temporal power spectra with a linear regression and found that the average log–log slope in the spatial domain was -2.40 and the average slope in the temporal domain was -1.54 (Fig. 4B and D, green circles).

Substantially less is known about spatiotemporal correlations in underwater habitats. One previous study examined the spatial frequency spectrum of a sample of oceanic aquatic images as compared to a terrestrial set and found that the best fitting slope was relatively steeper in the aquatic imagery^35^. This finding indicates a relative lack of high spatial frequencies in the aquatic visual environment. The spatial power spectra we observed in the zebrafish habitats were consistent with this prior work (Fig. 4A and B, blue): the slope of the aquatic power spectra tended to be steeper than the terrestrial scenes (M_T_ =  -2.40, M_A_ =  -2.83, d′ = 1.56, p ≪ 0.001). Interestingly, we observed the opposite pattern for the slope of the temporal power spectra (Fig. 4C,D): the average temporal slope was shallower in the aquatic samples as compared to the terrestrial samples (M_T_ =  -1.54, M_A_ =  -1.32, d′ = 0.58, p = 0.027). These data suggest that the zebrafish habitat has higher spatial correlations but lower temporal correlations than the comparison terrestrial habitats.

Previous research has suggested that the spatiotemporal power spectra of terrestrial videos are well-predicted by relatively weak assumptions about the distribution of speeds that objects tend to move in the world^55^. Figure 4E and F show the median joint spatiotemporal spectra from our aquatic and terrestrial samples. Both spectra have a concentration of power at the lowest spatiotemporal frequencies, with a roughly monotonic fall off along both dimensions. Examining the differences between the spectra (Fig. 4G), we observed an increase in power in the zebrafish habitat at relatively low spatial frequencies and relatively high temporal frequencies, as expected from the marginal distributions. However, this analysis also suggests a source for this difference: the increase in power appears to fall along a regime that corresponds to fast speeds (iso-speed lines are illustrated as contours on the plot, and increase from the upper left to the lower right). Thus, the differences in spatiotemporal power might primarily arise from more fast motion occurring in the aquatic samples, possibly due to drifting particulates, swimming fish, and flowing vegetation.

## Discussion

### Implications for studying behavior

A central finding from our analysis is that zebrafish habitats have a distribution of visual contrast that is quantitatively different from terrestrial habitats. The distributions are similar in shape, but their differences suggest that high contrast visual input is simply less likely in the zebrafish habitat. One previous study examined contrast in oceanic aquatic imagery and concluded that contrast values tended to be lower as compared to terrestrial imagery^35^. The authors posited that this difference arises due to greater light absorption and scattering underwater. However, the presence of consistent aquatic and terrestrial contrast differences was questioned in a meta-analysis that contained a broader sample of terrestrial habitats^58^. Ultimately, the specifics of the distribution of visual contrast in an animal’s habitat will be affected by a number of environmental properties that are challenging to predict from other data sources, motivating the value of directly examining the habitats where model organisms reside.

For much of the history of zebrafish behavioral science, common behavioral assays—such as the optomotor and optokinetic responses—have primarily been elicited in the laboratory with simple, high contrast and high coherence stimuli such as drifting gratings^59^. While such stimuli have led to significant advances in understanding visually-guided behavior, a key question moving forward is whether visually-guided behaviors in zebrafish can be better understood based on behavioral adaptations to a sparse contrast environment. Recent studies investigating decision making using optomotor swimming have shown that larval zebrafish can perceive sparse motion cues generated by low coherence random dot patterns^60,61^, showing that the zebrafish visual system is able to perceive motion cues even under demanding conditions. Future work could examine how a range of visually guided behaviors are driven by visually sparse stimulation with naturalistic spatiotemporal distributions.

Indeed, the results of our study complement a converging set of evidence suggesting that optomotor behaviors represent specific adaptations to environmental cues. We found that dark contrasts dominate in zebrafish habitats, particularly in the lower visual field. Dark contrasts were recently shown to play a key role in driving the optomotor response in zebrafish^13^. Combined with recent data showing that optomotor behavior in zebrafish is primarily elicited by motion in the lower visual field^15^, and that the lower field bias is well-predicted by the statistics of optic flow experienced during self-motion in the zebrafish habitat^16^, these data suggest that this behavior may be well-adapted to exploit both contrast and optical flow patterns in lower visual field in the natural habitat. In particular, we hypothesize bright to dark transitions in the lower visual field may provide the most prevalent, reliable information for detecting and counteracting undesirable self-motion (e.g., passive drift in water currents). Importantly, optomotor responses are present across terrestrial and aquatic species. By learning about natural visual statistics for the zebrafish, our analysis provides evidence to support the general hypothesis that optomotor responses are adapted for self-stabilization, and also supports the hypothesis that species-specific differences may arise due to differences in natural contrast and motion statistics across the visual field in different environments^16^. The present study’s measurements of the spatiotemporal visual demands imposed solely by the habitat provide a counterpart to our previous analysis of localized optic flow produced by the interplay of self-motion and the environment^16^. Defining the visual structure of the environment when zebrafish are relatively still is particularly important for the larval stage where swimming is highly discontinuous.

**Figure 5:**
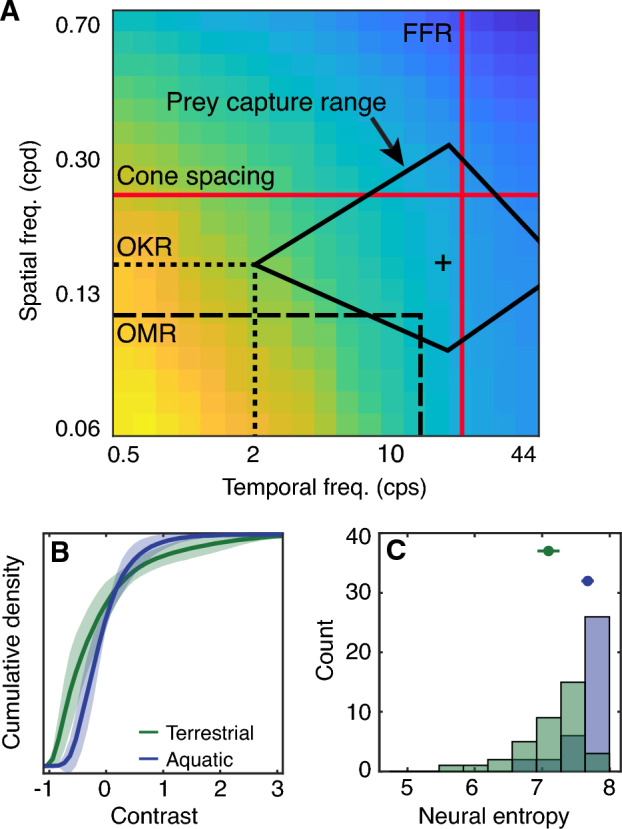
Implications for understanding visually guided behavior and neural coding. (**A**) The colormap shows the spatiotemporal power spectrum of the aquatic samples, reproduced from Fig. 4F using the same color scale. The horizontal dotted and dashed lines represent the spatial limit (visual acuity) of the optokinetic response (OKR) in larvae (0.16 cpd)^21^ and the optomotor response (OMR) in larvae (0.11 cpd, see our estimation based on^22^ in the methods section), respectively. The vertical dotted and dashed lines represent the approximate temporal limit of the OKR (~ 2 cps depending on stimulus velocity^20^) and the OMR (~ 14 cps^18,19^), respectively. Note that the temporal limit depends on the tested spatial frequencies and velocities. The solid black lines indicate the spatial and temporal stimulus bounds for larval prey capture (spatial: 0.09 to 0.33 cpd; temporal: 2 cps to 60 cps) and the cross indicates the "ideal" prey stimulus^25^. The red lines indicate the flicker fusion rate (FFR; 20 Hz)^26,27^ and the theoretical cone spacing limit (0.24 cpd) for larvae^21^. (**B**) Cumulative probability distributions for visual contrast are shown to illustrate different optimal neural response nonlinearities as described in^32^. (**C**) We applied each of the nonlinearities from (**B**) to the aquatic imagery in our dataset and computed the entropy of the predicted neural responses.

Looking beyond a specific behavior, we wanted to know how the limits of behavioral sensitivity broadly relate to the environmental power spectrum. To examine these relationships, in Fig. 5A we overlay key behavioral and retino-physiological limits for the larval zebrafish visual system over the spatiotemporal structure of the aquatic environment. Plotted behavioral parameters include the spatial (visual acuity) and temporal limits of the optokinetic response (OKR)^20,21^ and optomotor response (OMR)^18,19,22^ in larval fish, in addition to the spatial and temporal stimulus bounds for larval prey capture^25^. For reference, we also plot the flicker fusion rate (FFR)^26,27^ and the theoretical cone spacing limit for larvae^21^ as red lines. The spatial and temporal limits for these visually-driven behaviors appear to fall well within the spatiotemporal frequency regions with high power. The larval prey capture range is an exception to this observation: this higher-level behavior is likely adaptive for visual events that convey information of high behavioral importance (e.g., the presence of food), irrespective of their overall statistical likelihood across all visual imagery. While statistical descriptions of natural habitats are essential for understanding the demands placed on an animal’s sensory systems, an important next step in this line of research is to develop more quantitative models of optimal strategies for driving successful visually-guided behaviors within this habitat^15,62^. Our current results suggest that different visually-driven zebrafish behaviors, such as OMR and prey capture, exploit information in spatiotemporal regimes that vary substantially in their overall power, which can help inform our understanding of what types of situations these behaviors are adaptive for.

### Implications for neural coding

Behavioral adaptations to the natural habitat rely on neural adaptations that likely increase the efficiency of coding across cells, circuits, and systems. For example, in a seminal study, Laughlin suggested that the optimal contrast response nonlinearity for individual neurons has the shape of the cumulative probability distribution of contrast in the animals’ visual environment^32^. Such a nonlinearity lets all response levels be used equally and can thus maximize the neurons’ response entropy and information capacity. A pertinent previous study investigated potential changes in the response nonlinearities of bipolar retinal cells in salamanders that transition from a juvenile aquatic phase and an adult land phase^58^. The authors did not find evidence for any change in the bipolar cell responses accompanying the change in habitat, however, the relevant environmental contrast distributions were not directly measured, and the nonlinearities during both phases were well-matched to an overall theoretical optimum.

According to our dataset and the framework set out in^32^, if zebrafish visual neurons have adapted to the relatively narrow coding demand for contrast of their aquatic environment, we would expect the contrast response functions of visual neurons to exhibit a similarly narrow band of coding capacity (Fig. 5B, blue line) as compared to encoders of terrestrial contrast (Fig. 5B, green line). To quantify the differences between these response nonlinearities, we applied the two average nonlinearities shown in Fig. 5B to all our aquatic environment samples and computed the resulting entropy of the neural response distributions. As expected, applying the aquatic nonlinearity resulted in significantly higher neural entropy than applying the terrestrial nonlinearity to the same samples (Fig. 5C, M_T_ = 7.1 ± 0.49, M_A_ = 7.7 ± 0.28; t(70) = 6.13, d′ = 1.44, p ≪ 0.001). A caveat is that this approach assumes noise is additive and uniformly distributed, which is not necessarily the case in neural systems^63^, and the contrast transformation could be embodied by a range of different levels of visual processing. For example, nonlinearities may accumulate across the visual hierarchy, or may be distributed within a given population via heterogeneous neuronal types^63–65^.

Asymmetries between bright and dark contrasts in natural environments have also been extensively linked to adaptations in neural cells and circuits. In particular, anatomical and functional differences between the ON and OFF visual pathways in terrestrial animals are hypothesized to reflect adaptations to exploit the dark dominance of natural terrestrial imagery^37,38,43,44^. The current data suggest that neural adaptations exploiting a preponderance of dark contrasts in visual inputs should be advantageous for neural coding of visual information in the zebrafish habitat as well^14^. However, similar to chromatic vision, our data suggest that such adaptations may vary systematically across the visual field^12,52^. Interestingly, a recent report suggests that retinal positions of ON retinal ganglion cells are biased to the upper visual field, while the OFF retinal ganglion cells are biased to the lower visual field^52^. This reported asymmetry in retinal encoding maps onto our observed spatial contrast distribution, suggesting that the reported retinal ganglion cell distribution is well-adapted to encode the natural contrast statistics reported here. These anatomical findings also suggest a potential neural mechanism for the behavioral visual field biases discussed in the previous section with respect to optomotor behaviors. It would be informative to explore whether neural coding of higher-order motion (e.g.,^66^) differs between neurons in the zebrafish visual system with receptive fields in the upper and lower visual field.

Lastly, from an efficient coding perspective, it has been proposed that an important function of populations of neurons in early stages of the visual system is to reduce, or whiten, spatiotemporal correlations to remove unneeded redundancy in the visual signals. For example, whitening can be accomplished via center surround receptive fields and inhibitory interactions. There is good empirical evidence to support these predictions across diverse terrestrial species^59,67–69^. The relatively low temporal correlations in the zebrafish habitat suggest that less temporal whitening is needed to efficiently encode the aquatic habitat, assuming similar noise properties. Experiments in the manner of^69^, combining viewing of natural videos with functional recording, could be used to examine whether and how different stages of the zebrafish visual system alter the temporal spectrum of incoming natural visual input. Regardless of the environmental source, the greater statistical independence of temporal input to the zebrafish visual system provides an interesting opportunity to examine the role of temporal whitening in a model organism that evolved in a habitat with temporal structure that diverges from terrestrial animal models.

## Limitations

Capturing a complete sampling of any animal’s natural habitat is a daunting task. Even within the same species, habitats can be diverse and can differ across developmental stages. For the current dataset, we made efforts to sample a diverse set of environments, however, more systematic sampling schemes could be used in the future to ensure good coverage of the visual diversity experienced by zebrafish. We did not use a high dynamic range camera, which limits our ability to examine highlights and lowlights in the environments, and we used a device with video compression which may bias spatiotemporal statistics. Importantly, any artifacts would likely be relatively similar across the terrestrial and aquatic samples. We found evidence of more motion in the aquatic samples, but one possibility is that this motion was—in part—caused by small camera motion in flowing water. All samples from both environments are included in the Supplemental Material, and an examination of these videos suggests that the terrestrial videos do have very little environmental motion as compared to the aquatic samples.

## Conclusion

We hope that these habitat measurements will serve to expand our knowledge on how ecological niches have shaped the function of visual circuits and the behaviors these circuits subserve. Our database of field recordings is also freely available for further analysis. Integration of our analyses with the extensive neurophysiological and behavioral tools in zebrafish provides an exciting opportunity for attaining a unique neuroethological perspective on vertebrate vision.

## Supplementary Information

Supplementary Information 1.

Supplementary Video 1.

Supplementary Video 2.

## Data Availability

The video dataset can be accessed at https://doi.org/10.5281/zenodo.7502451, and Matlab code for performing the reported analyses is available at https://github.com/eacooper/ZebrafishAquaticVisualStatsCode.
