# Supplementary material for: Spatiotemporal visual statistics of aquatic environments in the natural habitats of zebrafish
Source: Sci Rep. 2023 Jul 25;13:12028. doi: 10.1038/s41598-023-36099-z (PMC10368656; doi:10.1038/s41598-023-36099-z)
Supplement: Supplementary file 1 — Supplementary Information 1. [file 41598_2023_36099_MOESM1_ESM.pdf]

Site	Date (2019)	Location	GPS Coordinates	Habitat(# videos)
1	Oct 11	Ujankuri	26° 16′ 33.5″ N, 91° 31′ 53.1″ E	A(6), T(3)
2	Oct 14	Khalihamari	26° 18′ 44.7″ N, 91° 28′ 30.0″ E	A(4)
3	Oct 15	Tumprop	26° 03′ 07.1″ N, 92° 25′ 52.5″ E	A(2)
4	Oct 17	Niz Udalguri	26° 46′ 25.4″ N, 92° 07′ 22.7″ E	A(1),T(1)
5	Oct 18	Umsiang	26° 03′ 25.8″ N, 92° 09′ 26.6″ E	T(1)
6	Oct 19	Goldighala	26° 17′ 01.0″ N, 91° 26′ 59.8″ E	A(1), T(1)

Table S1. Temporal and geographic data for recording sites. Coordinates indicate Global Positioning System (GPS) coordinates. In the habitat column, we indicate whether recordings were made in aquatic habitats, terrestrial habitats, or both (A = aquatic, T = terrestrial) and the number of videos that were recorded in each habitat type.

Figure S1: Site descriptions

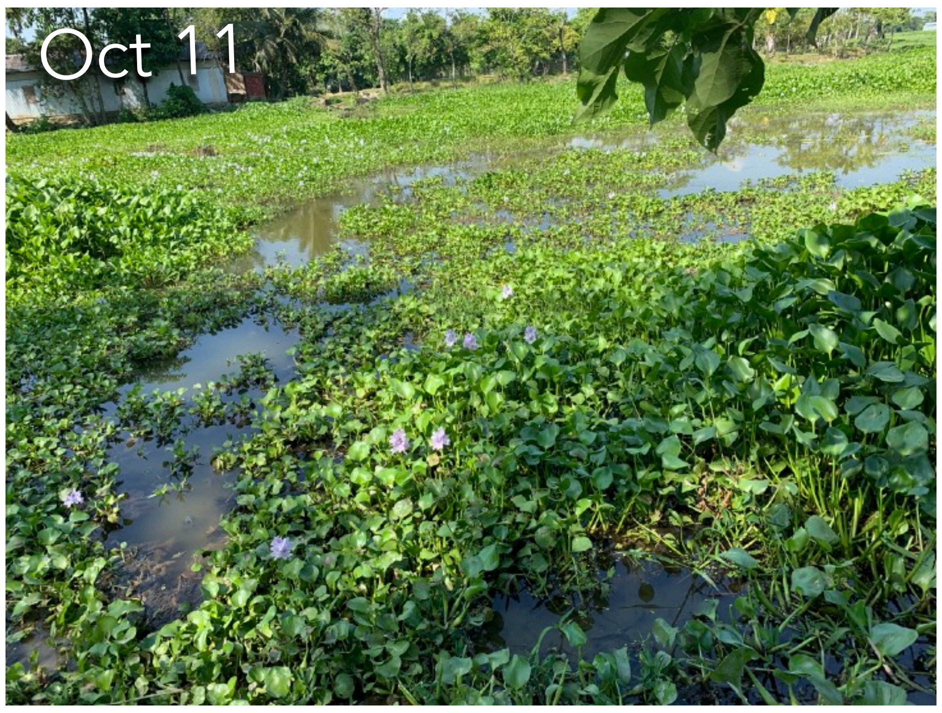

Ujankuri (26° 16′ 33.5″ N, 91° 31′ 53.1″ E): Floodplain wetland of the Brahmaputra River. Still water with a silt substrate. Filming was conducted between 8:00 - 10:00 in full sun, air temperature was 31C with no wind. (aquatic and terrestrial videos)

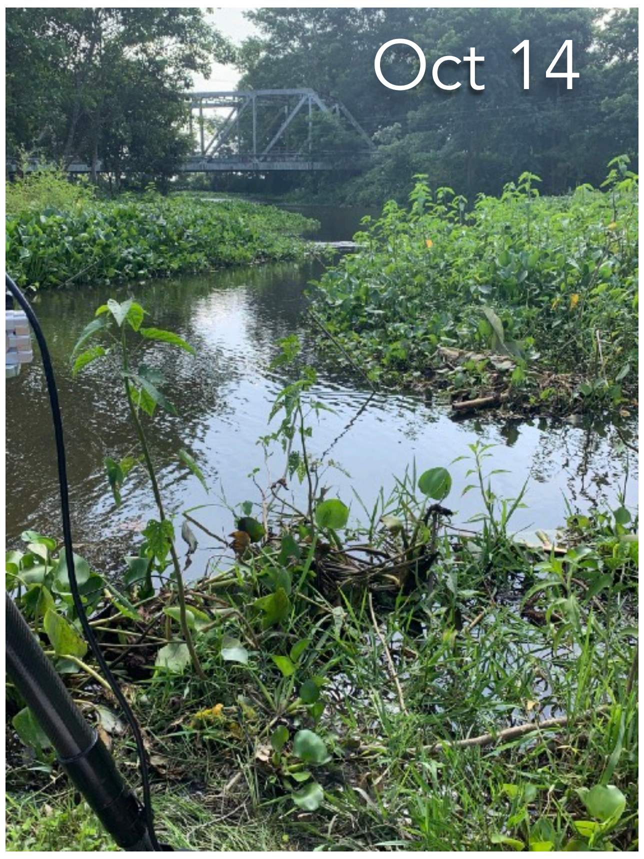

Khalihamari (26° 18′ 44.7″ N, 91° 28′ 30.0″ E): Second order stream with low flow conditions. Substrate type is silty with plant debris. Filming was conducted between 9:10 - 11:00 in full sun, air temperature was 31C with no wind. (aquatic videos only)

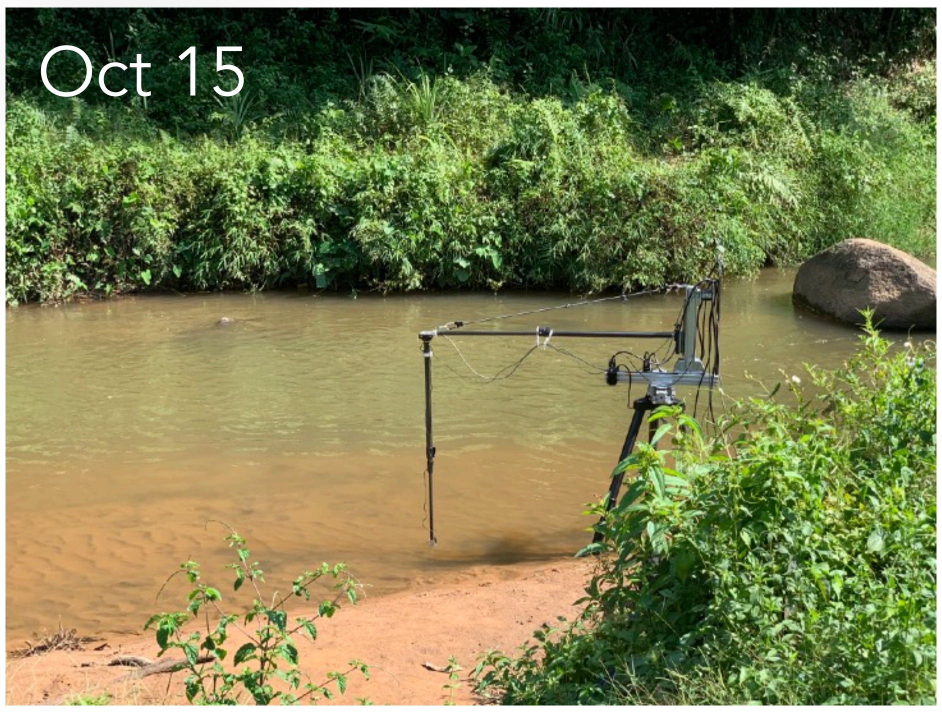

Tumprop (26° 03′ 07.1″ N, 92° 25′ 52.5″ E): Forested stream with a sandy substrate and moderate flow. Filming was conducted between 11:50 - 14:45 in full sun, air temperature was 33C with no wind. (aquatic videos only)

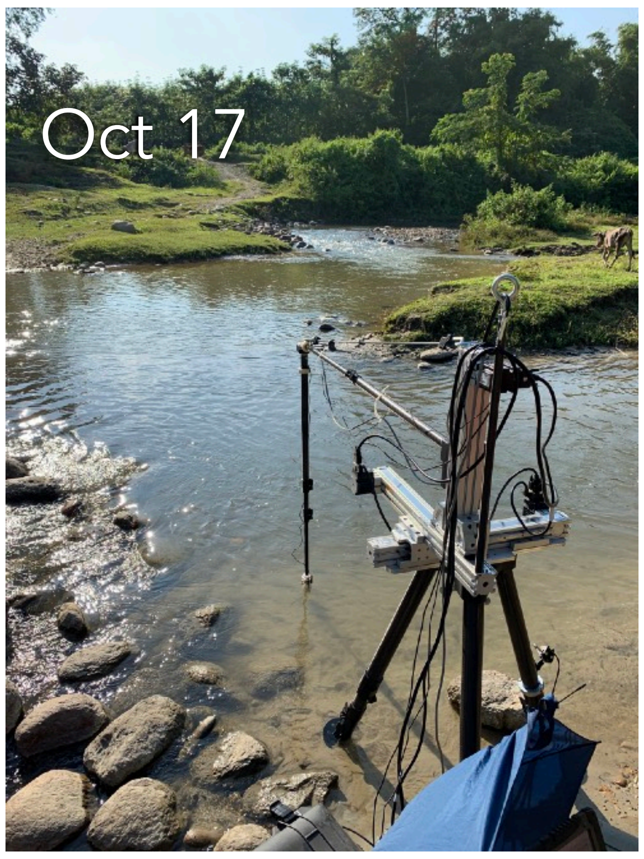

Niz Udalguri (26° 46′ 25.4″ N, 92° 07′ 22.7″ E): Third order stream with a sand and rock substrate and low flow. Filming was conducted between 14:00 and 16:20 in full sun, air temperature was 30C with no wind. (aquatic and terrestrial videos)

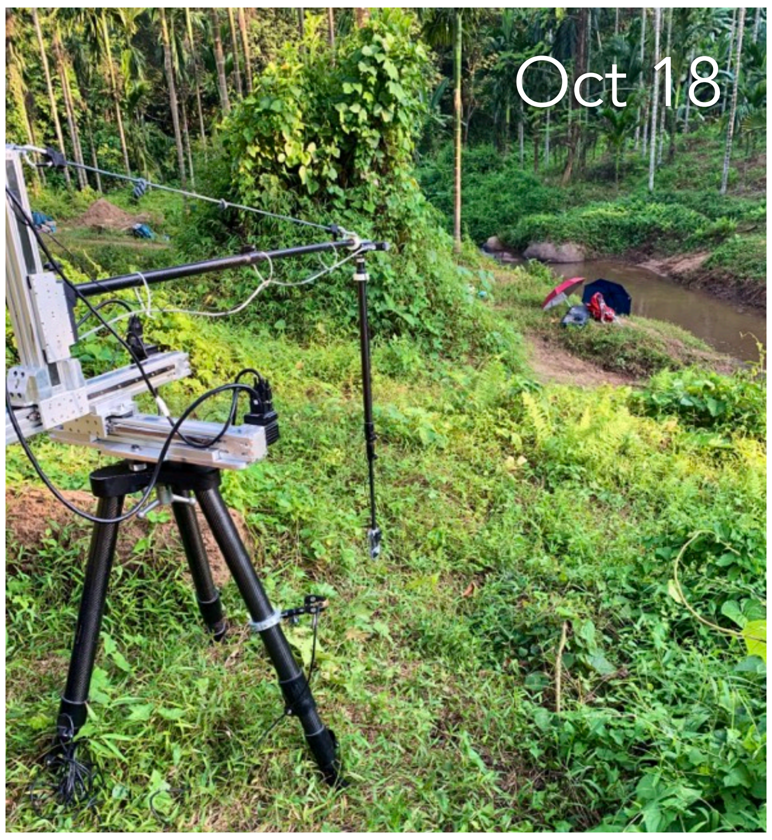

Umsiang (26° 03′ 25.8″ N, 92° 09′ 26.6″ E): Third order stream with a sand and pebble substrate and moderate flow. Filming was conducted between 13:20 and 15:15 in intermittent shade, air temperature was 31C with no wind. The water flow's force produced substantial camera vibrations. (terrestrial videos only)

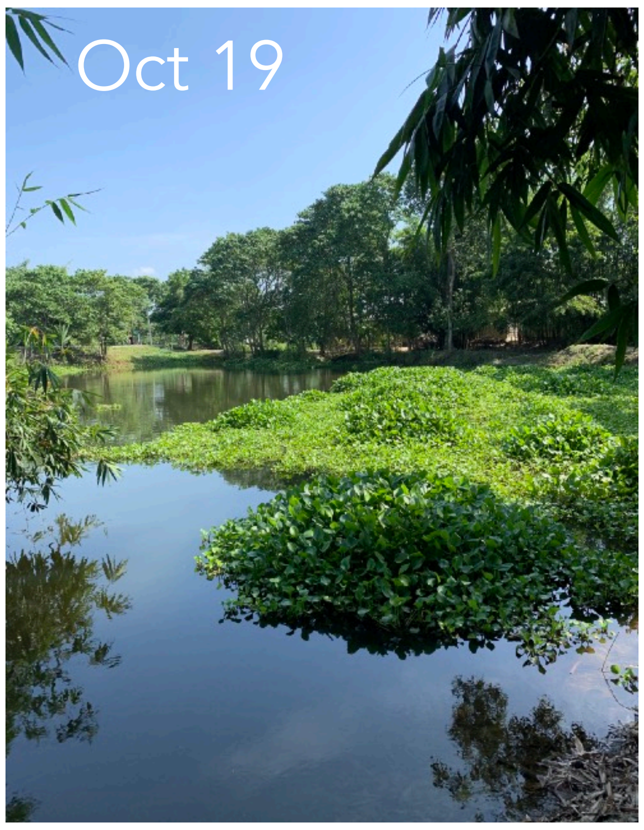

Goldighala (26° 17′ 01.0″ N, 91° 26′ 59.8″ E): Wetland area with still water and floating vegetation. Substrate type is silty with plant debris. This wetland area is surrounded by paddy fields. Filming was conducted between 9:20 and 12:00 in full sun with intermittent shade, air temperature was 29C with no wind. (aquatic and terrestrial videos)

Contrast					
	M_T	M_A	t-stat	d'	p
Variance	0.75 $\pm$ 0.32	0.23 $\pm$ 0.18	t(59) = 8.22	2.03	$\ll$0.001
Kurtosis	11.00 $\pm$ 10.00	4.50 $\pm$ 3.70	t(59) = 3.43	0.83	0.001
$M_T > 3$			t(24) = 3.78		$\ll$0.001
$M_A > 3$			t(35) = 2.39		0.023
Skewness	2.20 $\pm$ 1.10	1.00 $\pm$ 0.69	t(59) = 4.93	1.23	$\ll$0.001
$M_T > 0$			t(24) = 9.82		$\ll$0.001
$M_A > 0$			t(35) = 9.01		$\ll$0.001
Prop. negative	0.68 $\pm$ 0.10	0.60 $\pm$ 0.06	t(59) = 4.01	0.99	$\ll$0.001
$M_T > 0.5$			t(24) = 8.79		$\ll$0.001
$M_A > 0.5$			t(35) = 10.67		$\ll$0.001
Prop. neg. (local)	0.57 $\pm$ 0.02	0.55 $\pm$ 0.05	t(59) = 2.86	0.79	0.006
$M_T > 0.5$			t(24) = 17.14		$\ll$0.001
$M_A > 0.5$			t(35) = 5.82		$\ll$0.001
Entropy	6.60 $\pm$ 0.24	6.02 $\pm$ 0.50	t(59) = 5.30	1.46	$\ll$0.001
Power spectrum					
	M_T	M_A	t-stat	d'	p
Spatial slope	-2.40 $\pm$ 0.24	-2.83 $\pm$ 0.31	t(59) = 5.85	1.56	$\ll$0.001
Temporal slope	-1.54 $\pm$ 0.41	-1.32 $\pm$ 0.36	t(59) = -2.26	0.58	0.027

Table S2. T-tests examining terrestrial (T) and aquatic (A) visual environment differences. For each test, there are 25 terrestrial samples and 36 aquatic samples. Values in M_T and M_A columns indicate means and standard deviations for two-sample t-tests. Rows labeled $M_T > X$ indicate single-sample t-tests of the indicated mean (X).

Stats	Source	Sum Sq.	d.f.	Mean Sq.	F	η_p^2	Prob. > F
Mean	Environment	0.08	1	0.08	3.54	0.03	0.06
	Hemifield	13.18	1	13.18	565.13	0.83	$\ll$0.001
	Env. × Hemi.	0.72	1	0.72	30.93	0.21	$\ll$0.001
	Error	2.75	118	0.02			
	Total	16.06	121				
Variance	Environment	5.57	1	5.57	61.49	0.34	$\ll$0.001
	Hemifield	8.37	1	8.37	92.44	0.44	$\ll$0.001
	Env. × Hemi.	3.43	1	3.43	37.90	0.24	$\ll$0.001
	Error	10.68	118	0.09			
	Total	26.45	121				
Kurtosis	Environment	1310.57	1	1310.57	28.05	0.19	$\ll$0.001
	Hemifield	283.09	1	283.09	6.06	0.05	0.02
	Env. × Hemi.	216.12	1	216.12	4.62	0.04	0.03
	Error	5514.17	118	46.73			
	Total	7248.52	121				
Skewness	Environment	38.84	1	38.84	42.32	0.26	$\ll$0.001
	Hemifield	4.96	1	4.96	5.41	0.04	0.02
	Env. × Hemi.	2.39	1	2.39	2.60	0.02	0.11
	Error	108.32	118	0.92			
	Total	153.47	121				
Prop. negative	Environment	0.18	1	0.18	11.29	0.09	0.001
	Hemifield	6.78	1	6.78	420.29	0.78	$\ll$0.001
	Env. × Hemi.	0.07	1	0.07	4.45	0.04	0.04
	Error	1.90	118	0.02			
	Total	9.43	121				
Prop. neg. (local)	Environment	0.02	1	0.02	8.35	0.06	0.005
	Hemifield	0.002	1	0.002	0.64	0.01	0.43
	Env. × Hemi.	0.002	1	0.002	0.82	0.01	0.37
	Error	0.32	118	0.003			
	Total	0.34	121				

Table S3. ANOVAs examining terrestrial and aquatic visual environment differences in the upper and lower fields. Each section represents a 2x2 ANOVA examining main effects of environment (terrestrial vs. aquatic) and hemifield (upper versus low), as well as interactions.
